# Unenhanced CT-based predictive model to identify small bowel necrosis in patients with mechanical small bowel obstruction

**DOI:** 10.1186/s12880-023-01041-2

**Published:** 2023-06-12

**Authors:** Xianwei Liu, MingJie Zhu, Ming Wu, Zhangsong Cheng, Xiaoyu Wu, Renfang Zhu

**Affiliations:** 1 Department of General Surgery, Jiujiang No.1 People’s Hospital, Jiangxi province, Jiujiang, Jiujiang China; 2 Department of Gastrointestinal Surgery, The People’s Hospital of Leshan, Sichuan province, Leshan, China; 3 Department of Image Center, Jiujiang No.1 People’s Hospital, Jiangxi province, Jiujiang, China

**Keywords:** Mechanical small bowel obstruction, Small bowel necrosis, Unenhanced CT

## Abstract

**Objectives:**

To investigate the diagnostic value of unenhanced CT in mechanical small bowel obstruction (SBO) with small bowel necrosis, and to establish a predictive model.

**Methods:**

From May 2017 to December 2021, the patients with mechanical SBO admitted to our hospital were retrospectively collected. Taking pathology-confirmed small bowel necrosis as the gold standard, the experimental group was composed of patients with small bowel necrosis confirmed by pathology, and the control group was composed of patients with no intestinal necrosis confirmed by surgery or successful conservative treatment with no recurrence of intestinal obstruction during 1-month followed-up.

**Results:**

A total of 182 patients were enrolled in this study, 157 patients underwent surgery, of which 35 patients were accompanied with small bowel necrosis and 122 patients were not (33 patients with ischemic findings at surgery without necrosis). Finally, there were 35 patients in the experimental group and 147 patients in the control group. Multivariable logistic regression showed that increased attenuation of small bowel wall (*P* = 0.002), diffuse mesenteric haziness (*P* = 0.010), difference of CT value between mesenteric vessel and aorta (*P* = 0.025) and U-/C-shaped small bowel loop (*P* = 0.010) were independent risk factors for the diagnosis of mechanical SBO with small bowel necrosis. Through internal verification, the area under curve (AUC) of the predictive model reached 0.886 (95%CI: 0.824–0.947), and the calibration result was moderate.

**Conclusion:**

Multiple features (increased attenuation of small bowel wall; difference of CT values between mesenteric vessel and aorta; diffuse mesenteric haziness; and U-/C-shaped small bowel loop) of unenhanced CT have clinical value in the diagnosis of mechanical SBO with small bowel necrosis. The predictive model based on these four features could achieve satisfactory efficiency.

**Supplementary Information:**

The online version contains supplementary material available at 10.1186/s12880-023-01041-2.

## Introduction

Mechanical small bowel obstruction (SBO) was one of the main complications requiring emergency surgery, accounting for 20~50% of emergency surgeries [1]. Among them, about 60% of the incidence was caused by intestinal adhesion, especially after pelvic surgery, and most patients could be improved by medical care [2]. Mechanical SBO requiring surgery includes reversible ischemic changes and transmural necrosis of the small bowel [3]. Determining the occurrence of small bowel necrosis is an important indicator to judge the severity of intestinal obstruction and the indications of surgery [4]. Early and timely surgical intervention could reduce patients’ mortality and improve their prognosis [5]. Therefore, if intestinal necrosis could be accurately predicted, it would be beneficial to improve the clinical management of patients with mechanical SBO. However, at present, there is no a standardized and evidence-based criteria to judge small bowel necrosis [6]. In clinical practice, when a patient is diagnosed with signs of peritonitis through physical examination, the condition is often severe, and even accompanied with septic shock. The sensitivity and specificity of laboratory examination for diagnosing mechanical SBO with small bowel necrosis are relatively low [7].

Currently, computed tomography (CT) is the first choice for diagnosing intestinal obstruction [8], and is recommended by several studies [9–11]. However, most studies focused on enhanced CT [12], which may underestimate the diagnostic value of unenhanced CT [13]. Some studies had found that the absent or diminished enhanced of small bowel loop [13–15] and increased bowel wall attenuation in an isolated loop could be regarded as typical signs of small bowel necrosis in unenhanced CT [3, 16, 17]. However, these studies usually only paid attention to a few CT signs, and the sensitivity and specificity of the same signs in different literatures were quite different, and failed to establish a predictive model. In addition, some patients may not be suitable for enhanced CT because they are allergic to contrast media, or complicated with renal insufficiency and septic shock, etc. Therefore, the diagnostic value of enhanced CT for patients with mechanical SBO is better than that of unenhanced CT, but we still believe that the study of unenhanced CT also has important clinical value.

Therefore, our study aimed to find objective risk factors of mechanical SBO with small bowel necrosis on unenhanced CT imaging, and establish a predictive model.

## Materials and methods

### Design and patients

This single-center retrospective study was approved and waived the need for an informed consent by the medical ethics committee of our institution (JJSDYRMYY-YXLL-2021-258).

**Fig. 1:**
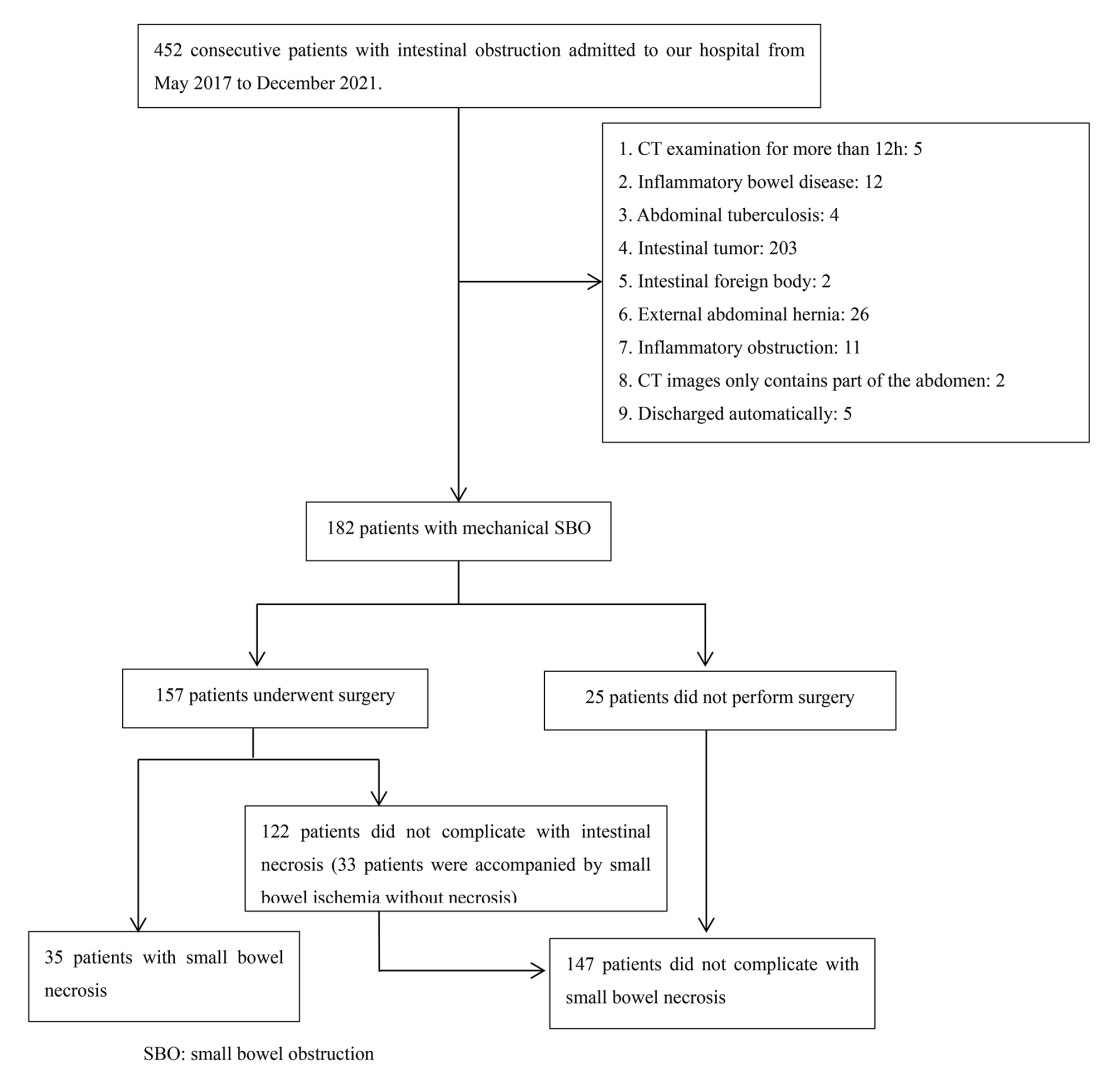
Flow chart of the study population

From May 2017 to December 2021, the patients with mechanical SBO admitted to our hospital were retrospectively collected, with “pathology-confirmed small bowel necrosis” as the gold standard. The experimental group consisted of patients with small bowel necrosis confirmed by pathology, while the control group consisted of patients with no small bowel necrosis confirmed by pathology or successful conservative treatment and without recurrence of intestinal obstruction in 1-month followed-up. To be included, patients must meet the following conditions: ⑴ adult patient (≥ 18 years); ⑵CT examination within 12 h after admission; ⑶ unenhanced CT scan was diagnosed as mechanical SBO. Patients who met the following conditions would be excluded: ⑴ non-adult patients; ⑵CT examination for more than 12 h or only enhanced CT, no unenhanced CT; ⑶small bowel obstruction caused by inflammatory bowel disease, abdominal tuberculosis, intestinal tumor (including peritoneal metastases), intestinal foreign body, and external abdominal hernia; ⑷ inflammatory obstruction; ⑸the CT imaging only contained part of the abdomen and the data collection could not be completed accurately; ⑹the patients were discharged automatically without treatment. Finally, we screened out 182 patients with mechanical SBO, and 157 patients underwent surgery, of which 35 patients were accompanied by small bowel necrosis and 33 patients were accompanied by small bowel ischemia without necrosis (Fig. 1). It should be noted that, except for 13 patients (six patients were allergic to contrast media, three patients had renal insufficiency, three patients had septic shock, and one patient refused enhanced CT because she was not sure whether he was allergic to contrast media.) who were not suitable for enhanced CT examination, the remaining 169 patients all underwent enhanced CT on the basis of unenhanced CT, but only the image data of unenhanced CT was extracted in this study. In addition, 157 patients in the control group were operated within 48 h of admission.

### Measuring instruments and parameters

Canon Medical AquiLion One TSX-305A320 slice helical CT was used for scanning, scanning parameters: voltage 120kv, current 500mas, pitch 0.813, rotation speed 0.5s, collimation width, and slice thickness 2-5 mm.

### Image analysis

**Fig. 2:**
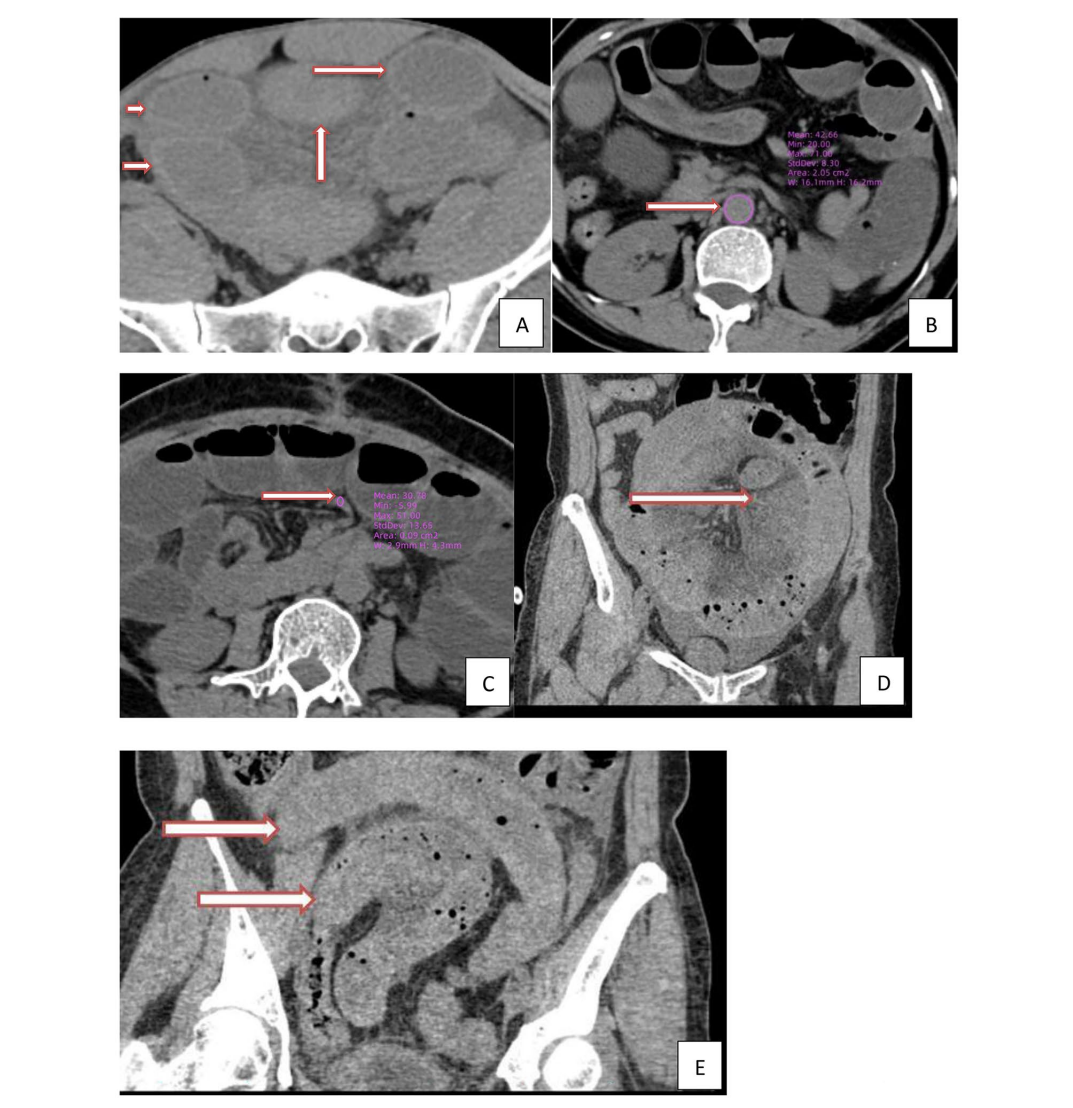
CT finding

CT images were analyzed by two radiologists (M.W. and ZS.C.) with 10 years of experience in abdominal CT, who were familiar with the standard of imaging indicators, and blinded to the status of patients (experimental or control), the final value of those imaging indicators were determined by a consensus read. A total of 17 imaging indicators were collected from 182 unenhanced CT data collected by the imaging workstation, which were defined as follows, and the schematic diagram was shown in Fig. 2 and supplementary materials.

The following imaging indicators of unenhanced CT were recorded:

(1) Increased attenuation of small bowel wall was subjectively defined as high density of the small bowel wall of a dilated loop compared with the healthy dilated loop [14, 18].

(2) CT value of small bowel wall was defined as the CT value of the axial plane where the intestinal obstruction was most obvious [18].

(3) Small bowel wall annular thickening was defined as annular wall thickness ≥ 2 mm [19].

(4) Small bowel lumen dilation was defined as the maximum diameter of the intestinal lumen at the most obvious dilatation was ≥ 3 cm [19].

(5) Maximum diameter of small bowel lumen dilation was defined as the maximum diameter of the intestinal lumen at the most obvious dilatation [19].

(6) Mesenteric ground glass sign was defined as the adipose tissue density around the blood vessels in the mesentery area was higher than that of the normal mesenteric fat, showing cloudy or ground glass-like changes [20].

(7) CT value of mesenteric vessel was defined as the CT value of the most obvious mesenteric vessels on the cross-section, most of the vessels were vein [21].

(8) Difference CT values between mesenteric vessel and aorta was defined as the CT value of mesenteric vessel minus the CT value of aorta.

(9) Mesenteric vasodilation was defined as relative dilatation of the mesenteric vessel around the small bowel obstruction site compared with those distant from the obstruction site [22].

(10) Diffuse mesenteric haziness was defined subjectively as diffuse density increase of mesenteric fat around the small bowel obstruction caused by edema [23].

(11) Small bowel fecal gas sign was defined as the presence of fecal material containing air bubbles in a single segment in the small bowel lumen proximal to the obstructive zone [24].

(12) U-/C-shaped small bowel loop was defined as the obstruction points at both ends of a dilated intestine, forming a “U”-shaped or “C”-shaped dilated intestinal loop, and the mesentery in the intestinal loop is stretched and unified gather between two obstruction points [25].

(13) Ascites was defined as intraperitoneal fluid visible to the naked eye on the cross-Sect.  [6]

(14) Peritoneal thickening was subjectively defined as the thickening of peritoneum around intestinal obstruction, paracolic sulcus or pelvis compared with normal peritoneum [26].

(15) Pneumatosis intestinalis was defined as the presence of gas in the intestinal wall [13].

(16) Portal venous gas was defined as the presence of gas in the portal vein [27].

(17) Whirlpool sign was defined as a soft tissue mass with internal structures of swirling mesenteric vessels and fat attenuation reflecting mesenteric torsion [28].

### Statistical analysis

Categorical data are presented as number and percentage and were compared by Pearson’s chi-square test or Fisher’s exact test. When continuous variables conformed to normal distribution, variables were expressed as mean ± standard deviation and compared using t test; when they conformed to non-normal distribution, variables were expressed as median sum (range) and compared using Wilcoxon test.

Inter observer variability assessment was used Cohen kappa test for categorical variables and intraclass correlation coefficient (ICC) for continuous variables [29]. The 95% confidence intervals (CI) were reported for each ICC. The interpretation of kappa values just like as Landis and Koch classification [30] (0.00, poor agreement; 0.00–0.20, slight agreement; 0.21–0.40, fair agreement; 0.41–0.60, moderate agreement; 0.61–0.80, good agreement, and 0.81–1.00, excellent agreement). The interpretation of ICC was described as follow [31]: < 0.5, poor agreement; 0.5–0.75, moderate agreement; 0.75–0.9, good agreement, and > 0.90, excellent agreement. Variables with kappa values < 0.61 and ICC < 0.75 were excluded for further analysis. The stepwise selection was used to establish a multivariate logistic regression model to determine the variables independently associated with small intestinal necrosis.

All data were analyzed using SPSS 23 Statistics software (version 23; IBM, Armonk, NY), and *P*<0.05 was determined to be statistically significant. We developed the predictive nomogram by R 4.2.1 (http://www.r-project.org) with the rms package. C-statistic was adopted to validate the nomogram internally. To reduce overfit bias, we validated the nomogram internally by bootstraps with 1000 resamples.

## Results

### Study population

The general characteristics of the study population were presented in Table 1. A total of 182 patients were enrolled in this study, 157 patients underwent surgery, of which 35 patients were accompanied with small bowel necrosis and 122 patients were not (33 patients with ischemic findings at surgery without necrosis). Finally, there were 35 patients in the experimental group and 147 patients in the control group (Fig. 1). The causes of their mechanical SBO were presented in Table S1. There were no statistically significant differences in age, gender, and body mass index (BMI) between the two groups, but patients without history of abdominal surgery (42.9% vs. 14.1%, p < 0.001) were more likely to accompany with small bowel necrosis.

**Table 1:** The general characteristics of the study population

		Experimental group	Control group	*p*
All		35	147	
Age(year)		57.8 ± 18.2	51.2 ± 18.1	0.054^α^
Gender	Male	22(62.9%)	78(53.1%)	0.295^β^
	Female	13(37.1%)	69(46.9%)	
BMI		20.0(16.2–32.9)	20.1(12.5–31.2)	0.814^γ^
History of abdominal surgery	Yes	20(57.1%)	122(85.9%)	0.001^β^
	No	15(42.9%)	25(14.1%)	

BMI: Body Mass Index; α: t test; β: Chi-square test; γ: Wilcoxon test

### Unenhanced CT finding

The results of the consensus reading and inter observer agreements were presented in Table 2. Except for the CT value of small bowel wall (ICC=0.725; 95% CI=0.649–0.788), other indicators were all in good agreement (Table 2). In addition, portal venous gas was found in only one patient. Therefore, CT value of small bowel wall and portal venous gas were excluded from subsequent analysis.

**Table 2:** The results of the inter observer agreement of 17 unenhanced CT imaging indicators by two imaging radiologists

CT findings	Inter observer agreement
Increased attenuation of small bowel wall	0.908^*^
CT value of small bowel wall	0.725 (0.649–0.788)^#^
Small bowel wall annular thickening	0.959^*^
Small bowel lumen dilation	0.954^*^
Maximum diameter of small bowel lumen dilation	0.854 (0.809–0.889)^#^
Mesenteric ground glass sign	0.839 ^*^
CT value of mesenteric vessel	0.885 (0.849–0.913)^#^
Difference of CT value between mesenteric vessel and aorta	0.835 (0.785–0.874)^#^
Mesenteric vasodilation	0.792^*^
Diffuse mesenteric haziness	0.884^*^
Small bowel fecal gas sign	0.849^*^
U-/C-shaped small bowel loop	0.938^*^
Ascites	1^*^
Peritoneal thickening	0.967^*^
Pneumatosis intestinalis	0.854^*^
Portal venous gas	1^*^
Whirlpool sign	0.788^*^

Inter observer variability assessment was used Kappa test for categorical variables (^*^), and intraclass correlation coefficient (ICC) for quantitative variables (^#^). ICC were reported with their 95% confidence intervals in parentheses. SBO: small bowel obstruction, CT: Computed tomography

There were statistically significant differences in difference CT values between mesenteric vessel and aorta (41.8 ± 4.9 vs. 45.3 ± 6.2, *P* = 0.001), increased attenuation of small bowel wall (65.7% vs. 22.4%, *P* < 0.001), small bowel wall annular thickening (51.4% vs. 22.4%, *P* = 0.001), mesenteric ground glass sign (97.1% vs. 78.2%, *P* = 0.009), diffuse mesenteric haziness (80.0% vs. 27.2%, *P* < 0.001), U/C-shaped small bowel loop (62.9% vs. 24.5%, *P* < 0.001), ascites (91.4% vs. 54.4%, *P* < 0.001) and peritoneal thickening (77.1% vs. 46.3%, *P* = 0.001) between the experimental and control group (Table 3). The rest indicators showed no significant differences (Table 3).

**Table 3:** Comparison of imaging data between two groups

Imaging data	No./Y or N	Experimental group	Control group	*p*
Maximum diameter of small bowel lumen dilation(cm)	170	4.1(2.8–6.9)	4.4(2.5–7.6)	0.922^γ^
CT value of mesenteric vessel (Hu)	182	49.2 ± 14.0	46.0 ± 9.8	0.124^α^
Difference of CT values between mesenteric vessel and aorta (Hu)	182	41.8 ± 4.9	45.3 ± 6.2	0.001^α^
Increased attenuation of small bowel wall	Y	23(65.7%)	33(22.4%)	< 0.001^β^
	N	12(34.3%)	114(77.6%)	
Small bowel wall annular thickening	Y	18(51.4%)	33(22.4%)	0.001^β^
	N	17(48.6%)	114(77.6%)	
Small bowel lumen dilation	Y	33(94.3%)	137(93.2%)	0.816^δ^
	N	2(5.7%)	10(6.8%)	
Mesenteric ground glass sign	Y	34(97.1%)	115(78.2%)	0.009^δ^
	N	1(2.9%)	32(21.8%)	
Mesenteric vasodilation	Y	10(28.6%)	46(31.3%)	0.754^β^
	N	25(71.4%)	101(68.7%)	
Diffuse mesenteric haziness	Y	28(80%)	40(27.2%)	<0.001^β^
	N	7(20%)	107(72.8%)	
Small bowel fecal gas sign	Y	14(40%)	40(27.2%)	0.137^β^
	N	21(60%)	107(72.8%)	
U-/C-shaped small bowel loop	Y	22(62.9%)	36(24.5%)	<0.001^β^
	N	13(37.1%)	111(75.5%)	
Ascites	Y	32(91.4%)	80(54.4%)	<0.001^δ^
	N	3(8.6%)	67(45.6%)	
Peritoneal thickening	Y	27(77.1%)	68(46.3%)	0.001^δ^
	N	8(22.9%)	79(53.7%)	
Pneumatosis intestinalis	Y	2(5.7%)	1(0.7%)	0.095^β^
	N	33(94.3%)	146(99.3%)	
Whirlpool sign	Y	5(14.3%)	16(10.9%)	0.571^β^
	N	30(85.7%)	131(89.1%)	

No.: number; Y: yes; N: no; α: t test; β: chi-square test; γ: Wilcoxon test; δ: Fisher’s exact test

### Risk factors for mechanical SBO with intestinal necrosis

The variables with *P* < 0.1 in univariable logistic regression were summarized and multivariable logistic regression was performed. Among them, there were four indicators of increased attenuation of small bowel wall (*P* = 0.002, OR = 5.778, 95% CI: 1.895–17.618), difference CT values between mesenteric vessel and aorta (*P* = 0.025, OR = 0.899, 95% CI: 0.819–0.986), diffuse mesenteric haziness (*P* = 0.010, OR = 4.649, 95% CI: 1.450-14.905), and U-/C-shaped small bowel loop (*P* = 0.010, OR = 4.105, 1.398–12.053) were independent risk factors for the diagnosis of mechanical SBO with small bowel necrosis (Table 4).

**Table 4:** Logistic regression statistics for general characteristics and imaging data

Variables	Univariate analysis			Multivariate analysis		
	*P*	OR	95% CI	*P*	OR	95% CI
Gender	0.297	1.497	0.701–3.196			
Age	0.056	1.021	0.999–1.044	0.850	1.003	0.973–1.033
BMI	0.327	1.055	0.948–1.174			
History of abdominal surgery	< 0.001	0.217	0.098–0.478	0.060	0.353	0.119–1.045
Increased attenuation of small bowel wall	<0.001	6.621	2.980–14.710	0.002	5.778	1.895–17.618
Small bowel wall annular thickening	0.001	3.658	1.697–7.882	0.789	1.165	0.280–3.575
Small bowel lumen dilation	0.816	1.204	0.252–5.760			
Maximum diameter of Small bowel lumen dilation	0.921	0.980	0.652–1.471			
Mesenteric ground glass sign	0.030	9.461	1.247–71.805	0.718	0.640	0.057–7.180
Mesenteric vascular density	0.118	1.026	0.994–1.059			
Difference of CT values between mesenteric vessel and aorta	0.003	0.898	0.836–0.964	0.025	0.899	0.819–0.986
Mesenteric vasodilation	0.754	0.878	0.390–1.978			
Diffuse mesenteric haziness	<0.001	10.700	4.331–26.433	0.010	4.649	1.450-14.905
Small bowel fecal gas sign	0.140	1.783	0.828–3.842			
U-/C-shaped small bowel loop	< 0.001	5.218	2.387–11.405	0.010	4.105	1.398–12.053
Ascites	<0.001	8.933	2.619–30.476	0.131	3.071	0.716–13.173
Peritoneal thickening	0.002	3.921	1.671–9.201	0.999	0.999	0.218–3.140
Pneumatosis intestinalis	0.079	8.848	0.779-100.509	0.284	5.612	0.239–131.770
Whirlpool sign	0.573	1.365	0.464–4.017			

CI: confidence interval; CT: computed tomography; BMI: body mass index

### Establishment and validation of the predictive model

**Fig. 3:**
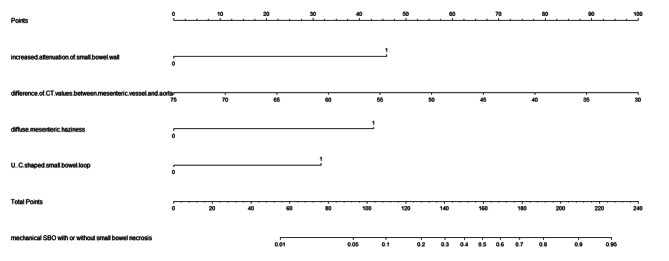
Nomogram for the predictive model of mechanical SBO with or without small bowel necrosis

**Fig. 4:**
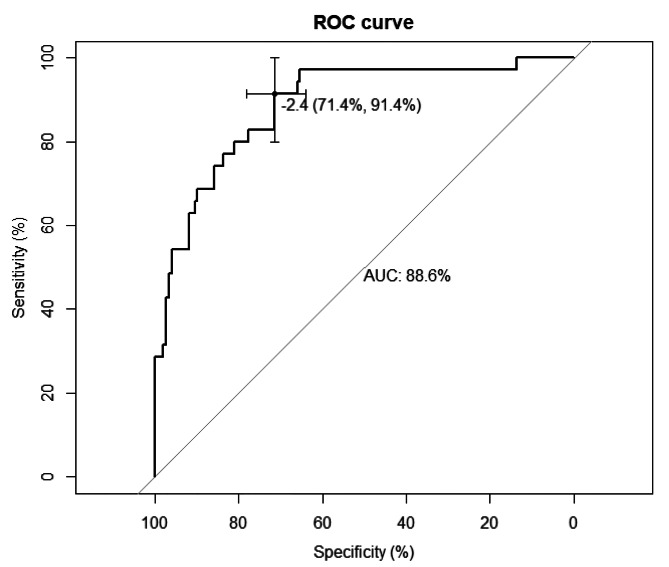
Receiver operating characteristic curve of the nomogram in the training dataset

**Fig. 5:**
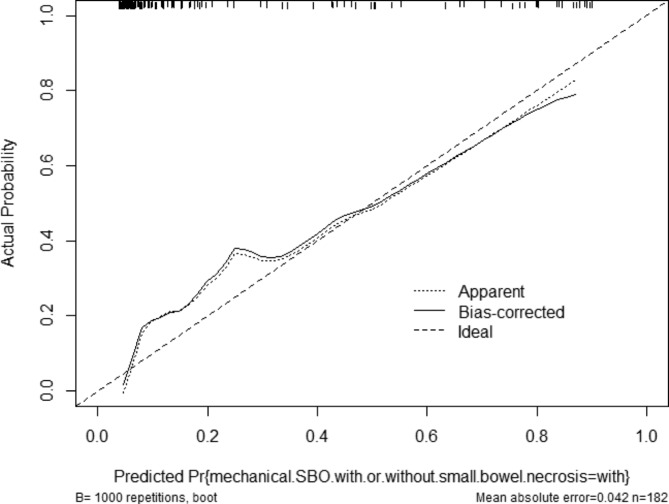
Calibration plot in the training dataset

Based on the results of multivariable logistic regression analysis, increased attenuation of small bowel wall, difference CT values between mesenteric vessel and aorta, diffuse mesenteric haziness, and U-/C-shaped small bowel loop were the most effective predictive factors for mechanical SBO with small bowel necrosis (Table 4). A nomogram was constructed by using these factors as shown in Fig. 3. The area under curve (AUC) of this nomogram reached 0.886 (95%CI: 0.824–0.947) through internal validation by bootstraps with 1000 resamples (Fig. 4). Calibration curve was shown in Fig. 5, which showed the predictive model was moderately calibrated.

## Discussion

Imaging diagnosis is of great significance to the treatment of patients with mechanical SBO. Chuong et al. [32] revealed that the combination of unenhanced and enhanced CT could improve the diagnostic efficiency of mechanical SBO. However, unenhanced CT is more economical, rapid and effective, and it can also avoid contraindications related to the enhanced CT. Our results also suggested that many features in unenhanced CT imaging had good diagnostic value for mechanical SBO with small intestinal necrosis, and the predictive model had achieved good AUC value.

Intestinal vascular circulation disorder could cause pathological changes of various tissues through various mechanisms (such as exudation, deterioration and proliferation), which could be reflected on CT imaging [33].

The exudation in different tissues presented different characteristics on CT imaging. (1) The exudation in intestinal wall was usually presented as a thickened, double-layered ring sign or target sign. Sometimes it could also cause oozing blood in the intestinal wall, which could be easily identified as a mixed high-density shadow on unenhanced CT imaging [18]. Unfortunately, the CT value of small-bowel wall failed to pass the ICC test, which should be related to the measurement of completely different areas of the small bowel wall by two radiologists. However, we still believe that the CT value of small-bowel wall would be a meaningful image indicator as long as we find a suitable measurement standard. (2) The exudation in mesentery was manifested as a cloudy water-like density which was usually distributed along the mesangial side of the involved intestinal wall [26, 34]. Corresponding features could be observed on CT imaging, such as mesenteric ground glass sign, mesenteric vasodilation, diffuse mesenteric haziness, change of CT value of mesenteric vessels, and whirlpool sign. In our study, the diffuse mesenteric haziness, and the difference of CT values between mesenteric vessel and aorta were all independent risk factors of mechanical SBO with small bowel necrosis. The mesenteric vasodilatation and whirlpool sign were not related to mechanical SBO with small bowel necrosis, which was consistent with previous studies [28, 35]. (3) The exudation in the intestinal lumen was presented as lumen effusion and expansion [4, 36], which were common CT signs of mechanical SBO, but not specificity in mechanical SBO with small bowel necrosis. In our study, there was no significant difference between the two groups in the small bowel lumen dilation and maximum diameter of small bowel lumen dilation. In addition to difference of exudation sites, difference of exudation components also presented different characteristics on CT imaging. When the exudative components were mainly liquid or protein, the CT value of ascites was often less than 10 Hu. If the CT value of ascites was above 10 Hu, it generally indicated co-infection, hemorrhagic effusions or necrosis [37]. In clinical practice, ascites could be easily obtained by paracentesis and had more objective results. Therefore, we did not measure the CT value of ascites. The results showed that the proportion of ascites in mechanical SBO patients with small bowel necrosis was higher, but there was not significant difference in multivariable logistic regression analysis.

In the process of metamorphism, according to the different stages of pathological changes, the intestinal wall shows successively thickening, weakening or disappearance of reinforcement, or thin paper-like shape [38]. In our study, increased attenuation of small bowel wall and small bowel wall annular thickening showed significant differences between the two groups and univariable logistic regression analysis. However, small bowel wall annular thickening may only be related to mechanical SBO with intestinal necrosis on unenhanced CT images, because this result had not been found in the current research on enhanced CT images [3, 25]. The increased attenuation of small bowel wall was also an independent predictor of mechanical SBO with small bowel necrosis. Previous literatures had shown that increased attenuation of small bowel wall was the most specific CT sign of intestinal ischemia [3, 39, 40]. Meanwhile, mucosal cells were sensitive to ischemia and hypoxia. Mucosal barrier dysfunction could lead to submucosal gas accumulation, gas-liquid inversion, submucosal gas accumulation and portal venous gas accumulation [41]. Small bowel fecal gas sign and pneumatosis intestinalis were caused by this reason, but they were not the risk factor of mechanical SBO with small bowel necrosis. Only one patient was identified with portal venous gas in our study. This is similar to the research of Lebert et al. [27]. The portal venous gas is very rare, and it is not specific for small bowel necrosis.

The CT findings of intestinal hyperplasia stage are thickening of intestinal wall and lead-like ankyloses, which usually indicates severe or intestinal ischemia [25]. U-/C-shaped small bowel loop signs were typical manifestations [42]. Our results also showed that U-/C-shaped small bowel loop was an independent risk factor of mechanical SBO with small bowel necrosis. As we all know, on unenhanced CT imaging, the signs of closed loop obstruction may predict small bowel necrosis better than the U-/C-shaped small bowel loop signs [43], but the signs of closed loop obstruction are usually accompanied by the U-/C-shaped small bowel loop in cross section, while the appearance of the U-/C-shaped small bowel loop does not represent closed loop obstruction [44]. Therefore, we finally chose the U-/C-shaped small bowel loop as one of the predictive indicators. Moreover, inflammatory factors released by necrotic intestinal wall could cause obvious peritoneal inflammation, leading to significant ascites, peritoneal thickening and fascia-like thickening [45]. In our study, there was a significant difference in peritoneal thickening between the two groups, but peritoneal thickening was not a risk factor in the logistic regression analysis.

Our study also found that mechanical SBO patients without history of abdominal surgery were more likely to be complicated with small bowel necrosis. This may be the deviation caused by our small case. Theoretically, the history of abdominal surgery is related to intestinal adhesion, and adhesion bands and internal hernias caused by intestinal adhesion are more likely to cause closed-loop intestinal obstruction, leading to small bowel necrosis [3, 22, 23] Nevertheless, this also gives a warning to gastrointestinal surgeons that mechanical SBO patients who had no history of abdominal surgery should also paid attention to in clinical practice.

Darras et al. [46] suggested that contrast-enhanced dual energy CT with virtual mono energetic image reconstruction at 70 keV maximizes the contrast to noise ratios of small bowel mural enhancement and could increase the diagnosis in assessing small bowel wall enhancement in patients with SBO. However, more scholars believed that dual energy CT may be more valuable in the diagnosis of acute mesenteric ischemia, rather than mechanical SBO [47]. Moreover, dual energy CT is expensive and not a routine examination item. The major advantage of this study was to measure the difference of CT values between mesenteric vessel and aorta, which was confirmed as an independent risk factor of mechanical SBO with small bowel necrosis. Through the vitro experiment, Kirchhof et al. [48] found that the CT value of normal human abdominal aorta blood is 35–50 Hu, and that of venous blood is 55 ± 5 Hu, and the higher the content of deoxyhemoglobin is, the higher the CT value. It was also believed that the changes of CT values on unenhanced CT could be used as evidence to suspect intravascular obstruction. Meanwhile, Morita et al. [49] used the CT value changes in cerebrovascular density on unenhanced CT to diagnose cerebrovascular diseases. We also found that the difference of CT values between the experimental group and the control group was statistically significant, but there was a lot of overlap, which may need more clinical studies to confirm. At the same time, we established a predictive model for mechanical SBO with small bowel necrosis, and obtained satisfactory AUC and moderate calibration results.

The limitations of this study were as follows: Firstly, the small sample size and high proportion of surgical patients may lead to potential bias. Secondly, the predictive model was only validated internally instead of externally. Thirdly, this study did not distinguish simple and complicated mechanical SBO. According to the classification of mechanical SBO, it would be better to distinguish simple and complicated mechanical SBO. However, it is always difficult to accurately distinguish them in clinical practice [50].

## Conclusion

Multiple features (increased attenuation of small bowel wall; difference of CT values between mesenteric vessel and aorta; diffuse mesenteric haziness; U-/C-shaped small bowel loop) of unenhanced CT have clinical value in the diagnosis of mechanical SBO complicated with small bowel necrosis. The predictive model for mechanical SBO achieved satisfactory efficiency.

## Electronic supplementary material

Below is the link to the electronic supplementary material.

Supplementary Material 1

## Data Availability

The datasets used and/or analyzed during the current study are available from the corresponding author on reasonable request.
