# Supplementary material for: Unenhanced CT-based predictive model to identify small bowel necrosis in patients with mechanical small bowel obstruction
Source: BMC Med Imaging. 2023 Jun 12;23:80. doi: 10.1186/s12880-023-01041-2 (PMC10262409; doi:10.1186/s12880-023-01041-2)
Supplement: Supplementary file 1 — Supplementary Material 1 [file 12880_2023_1041_MOESM1_ESM.docx]

**Supplementary materials**

Table S1 The causes of 182 patients with mechanical SBO

| Cause | Experimental group | | Control group | |
| --- | --- | --- | --- | --- |
|  | With HAS (n=20) | Without HAS (n=15) | With HAS (n=122) | Without HAS (n=25) |
| Simple adhesion |  |  | 71 |  |
| Torsion caused by adhesion | 9 |  | 25 |  |
| Adhesive band | 8 |  | 17 |  |
| Internal hernia | 3 |  | 9 |  |
| Small bowel torsion |  | 15 |  | 18 |
| Intussusception |  |  |  | 3 |
| Fecalith |  |  |  | 3 |
| Cocoon abdomen |  |  |  | 1 |

HAS：History of abdominal surgery

Schematic diagram and measurement standards of extra CT imaging feature.


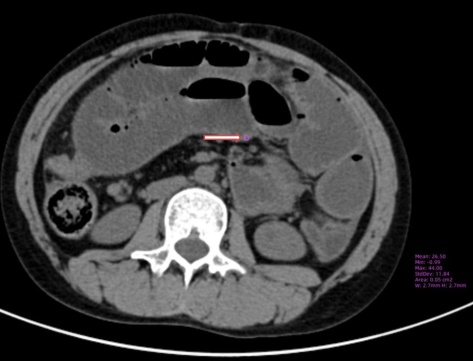


**A**

A: The arrow is where we measure the CT value of small bowel wall, with results shown in the upper-right corner. We selected the axial plane where the intestinal obstruction was most obvious. Referring to the relevant literature [1], the average of CT value of intestinal wall was measured by circling where the CT value of the intestinal wall increased most significantly, the edge of the circle should not exceed the intestinal wall, should keep a distance from high-density fecal shadows, air, and bone, to reduce the influence of partial volume effects on the measurement results. Unfortunately, it failed to pass the consistency test, which should be related to two radiologists measuring completely different areas of the small bowel wall. Therefore, we finally gave up this data.


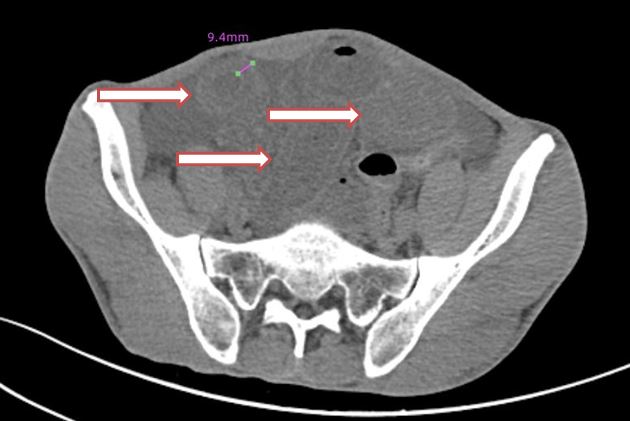


**B**

B: The arrow points to the annular thickening of the intestinal wall, with results shown in purple. The cross-section of the intestinal wall with the most obvious obstruction was selected. Referring to relevant literature[2], Annular wall thickness ≥2mm was considered positive, while ＜ 2mm or partial wall thickening (non-annular thickening) was considered negative.


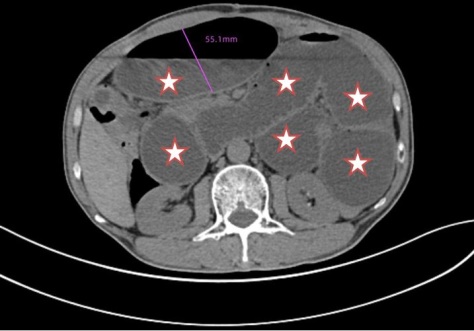


**C**

C: Small bowel lumen dilation (five-pointed star), the purple line is the maximum diameter of small bowel lumen dilation. The maximum diameter of the lumen dilatation was measured on the cross-section with most obvious intestinal obstruction. Referring to relevant literature[3], the maximum diameter of the intestinal lumen at the most obvious dilatation was measured. Patients with the maximum diameter of intestinal lumen dilatation was≥3cm, was judged as positive with intestinal lumen dilatation, with＜3cm was judged as negative.


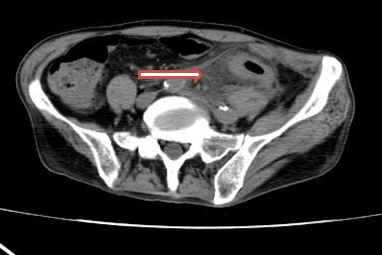


**D**

D: The arrow points to the mesenteric ground glass sign. Referring to relevant literature[4], the mesenteric ground glass sign was defined as: the adipose tissue density around the blood vessels in the mesentery area is higher than that of the normal mesenteric fat, showing cloudy or ground glass-like changes, which can be distinguished by the naked eye and regarded as positive.


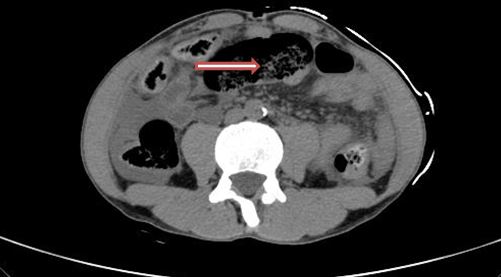


**E** abdominal aorta density measurement, a cross section was selected, a circular ROI area was drawn in the abdominal aorta, and the average density in the area was measured. It is best to keep a certain distance between the ROI area and the edge of the abdominal aorta to avoid the calcification of the aortic wall to reduce the partial volume effect. F

E: The arrow points to the small bowel fecal gas sign. Small bowel fecal gas sign refers to the presence of fecal material containing air bubbles in a single segment in the small bowel lumen proximal to the obstructive zone[5].


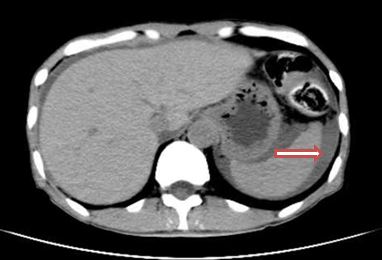


**F** abdominal aorta density measurement, a cross section was selected, a circular ROI area was drawn in the abdominal aorta, and the average density in the area was measured. It is best to keep a certain distance between the ROI area and the edge of the abdominal aorta to avoid the calcification of the aortic wall to reduce the partial volume effect. F

F: The arrow points to the ascites. Referring to the relevant literature [6], cross-sectional observation is usually selected, and intraperitoneal fluid can be judged as positive by the naked eye.


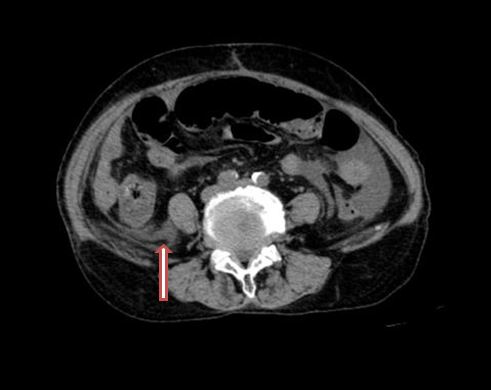


**G** abdominal aorta density measurement, a cross section was selected, a circular ROI area was drawn in the abdominal aorta, and the average density in the area was measured. It is best to keep a certain distance between the ROI area and the edge of the abdominal aorta to avoid the calcification of the aortic wall to reduce the partial volume effect. F

G: The arrow points to the thickened peritoneum. Referring to the relevant literature [7], cross-sections are usually selected to observe the peritoneum in the paracolic grooves, pelvis and other parts, especially the parts adjacent to the obstructed bowel segment. If the peritoneum is thickened by the naked eye, it will be judged as positive.


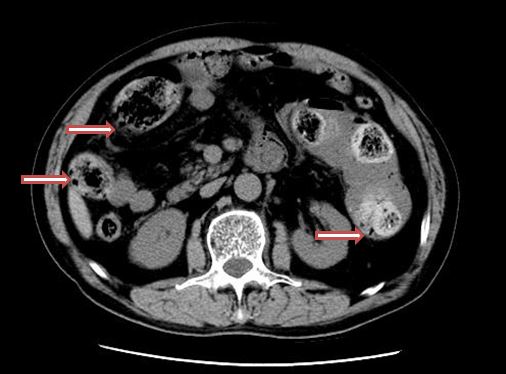


**H** abdominal aorta density measurement, a cross section was selected, a circular ROI area was drawn in the abdominal aorta, and the average density in the area was measured. It is best to keep a certain distance between the ROI area and the edge of the abdominal aorta to avoid the calcification of the aortic wall to reduce the partial volume effect. F

H: The arrow points to the pneumatosis intestinalis, referring to the relevant literature [8], a cross-section is usually selected. According to the characteristics of gas-liquid inversion and bead-like gas distribution, the presence of gas in the intestinal wall will be judged as positive.


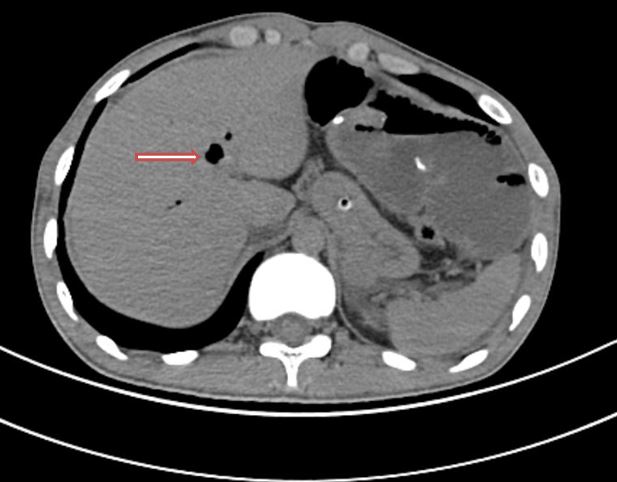


**I** abdominal aorta density measurement, a cross section was selected, a circular ROI area was drawn in the abdominal aorta, and the average density in the area was measured. It is best to keep a certain distance between the ROI area and the edge of the abdominal aorta to avoid the calcification of the aortic wall to reduce the partial volume effect. F

I: The arrow points to portal venous gas，referring to the relevant literature[9], cross-sectional observation is usually selected, and it is judged as positive if there is gas in the portal vein with naked eyes.


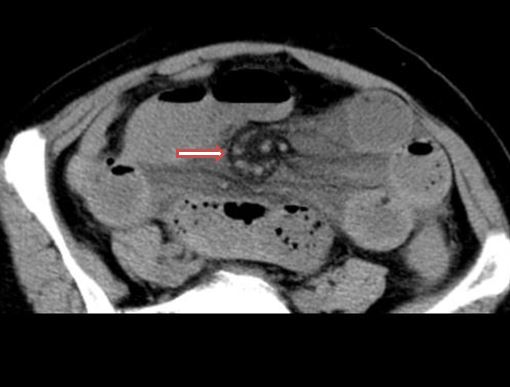


**J** abdominal aorta density measurement, a cross section was selected, a circular ROI area was drawn in the abdominal aorta, and the average density in the area was measured. It is best to keep a certain distance between the ROI area and the edge of the abdominal aorta to avoid the calcification of the aortic wall to reduce the partial volume effect. F

J: The arrow points to the whirlpool sign, which is defined as a soft tissue mass with internal structures of swirling mesenteric vessels and fat attenuation reflecting mesenteric torsion[10]. If the mesenteric torsion exceeds 360° it will be judged as a positive whirlpool sign combined with the criteria of relevant literature.

**References**

1. Kohga A, Kawabe A, Yajima K, Okumura T, Yamashita K, Isogaki J, Suzuki K, Muramatsu K: **CT value of the intestine is useful predictor for differentiate irreversible ischaemic changes in strangulated ileus**. *Abdominal radiology (New York)* 2017, **42**(12):2816-2821.

2. Zalcman M, Sy M, Donckier V, Closset J, Gansbeke DV: **Helical CT signs in the diagnosis of intestinal ischemia in small-bowel obstruction**. *AJR American journal of roentgenology* 2000, **175**(6):1601-1607.

3. Scaglione M, Romano S, Pinto F, Flagiello F, Farina R, Acampora C, Romano L: **Helical CT diagnosis of small bowel obstruction in the acute clinical setting**. *European journal of radiology* 2004, **50**(1):15-22.

4. Hayakawa K, Tanikake M, Yoshida S, Yamamoto A, Yamamoto E, Morimoto T: **CT findings of small bowel strangulation: the importance of contrast enhancement**. *Emergency radiology* 2013, **20**(1):3-9.

5. Khaled W, Millet I, Corno L, Bouley-Coletta I, Benadjaoud MA, Taourel P, Zins M: **Clinical Relevance of the Feces Sign in Small-Bowel Obstruction Due to Adhesions Depends on Its Location**. *AJR American journal of roentgenology* 2018, **210**(1):78-84.

6. Ten Broek RPG, Krielen P, Di Saverio S, Coccolini F, Biffl WL, Ansaloni L, Velmahos GC, Sartelli M, Fraga GP, Kelly MD *et al*: **Bologna guidelines for diagnosis and management of adhesive small bowel obstruction (ASBO): 2017 update of the evidence-based guidelines from the world society of emergency surgery ASBO working group**. *World journal of emergency surgery : WJES* 2018, **13**:24.

7. Thornton E, Mendiratta-Lala M, Siewert B, Eisenberg RL: **Patterns of fat stranding**. *AJR American journal of roentgenology* 2011, **197**(1):W1-14.

8. Sheedy SP, Earnest Ft, Fletcher JG, Fidler JL, Hoskin TL: **CT of small-bowel ischemia associated with obstruction in emergency department patients: diagnostic performance evaluation**. *Radiology* 2006, **241**(3):729-736.

9. Lebert P, Ernst O, Zins M, Lanchou M, Nzamushe JR, Vermersch M: **Pneumatosis intestinalis and portal venous gas in mechanical small bowel obstruction: Is it worrisome?** *Diagn Interv Imaging* 2021, **102**(9):545-551.

10. Mitsuyoshi A, Tachibana T, Kondo Y, Momono T, Aoyama H: **What We Can Learn from Cases of Synchronous Acute Mesenteric Obstruction and Nonocclusive Mesenteric Ischemia: How to Reduce the Acute Mesenteric Ischemia-Related Mortality Rate**. *Annals of vascular surgery* 2016, **32**:133.e111-137.

G

F
